# Public health concerns about Tuberculosis caused by Russia/Ukraine conflict

**DOI:** 10.1002/hsr2.1218

**Published:** 2023-04-17

**Authors:** Innocent K. Paul, Goodluck Nchasi, Deusdedith B. Bulimbe, Meshack K. Mollel, George G. Msafiri, Alex Mbogo, Mayunga B. Mswanzari, Msengi Joseph, Ashraf Mahmoud, Anastasiia Volkova

**Affiliations:** ^1^ Department of Medicine Catholic University of Health and Allied Sciences Mwanza Tanzania; ^2^ Department of Medicine and Dentistry The University of Dodoma Dodoma Tanzania; ^3^ Department of Medicine, School of Medicine Muhimbili University of Health and Allied Sciences Dar es Salaam Tanzania; ^4^ Department of Medicine Kilimanjaro Christian Medical University College Kilimanjaro Tanzania; ^5^ Department of Medicine, Faculty of Medicine Lviv National Medical University Lviv Oblas Ukraine

**Keywords:** public health, Tuberculosis, Ukraine

## Abstract

According to WHO, Ukraine has the fourth‐highest Tuberculosis (TB) incidence in the WHO European region while globally has the fifth‐highest number of confirmed cases of extensively drug‐resistant TB. Before the Russian invasion in Ukraine several interventions have been employed to mitigate the TB epidemic in the country. However, the ongoing war has demolished meticulous efforts and subsequently worsen the situation. WHO in collaboration with the Ukraine government and other organizations such as EU and UK are required to take up arms against the situation. In this work, implications brought up from the war, efforts, and recommendations to battling TB epidemic due to the war are highlighted.

## 1 INTRODUCTION

In any nation, peace is crucial in ensuring the economy's stability, political endurance, and even the overall public health of its citizens. Considering the Russia/Ukraine conflict, which began on February 24, 2022. The Russian invasion resulted in physical destruction and a myriad of public health impacts in Ukraine, considering that it is one of the prioritized nations with a high burden of Tuberculosis in the WHO European region and globally.^1^, ^2^ Moreover, Ukraine faces a high burden of rifampicin‐resistant or multidrug‐resistant TB (RR/MDR‐TB).^1^

Upon the conflict, many Ukrainians have been forced to migrate to neighboring countries away from the war that is still going on in their country. Since neither ECDC nor WHO guidelines recommend universal testing of TB infection of refugees fleeing to the European countries, it is an undeniable truth that the progression and transmission of the RR/MDR‐TB strains caused by Ukrainian migrants will be at a high rate that can greatly affect the public health status of nations receiving Ukrainian refugees.^1^

As of October 2022, more than 7 million refugees have fled from Ukraine to Hungary, Poland, Romania, the Czech Republic, and other European nations.^3^ This large movement of people poses a great risk to Europe due to Ukraine's high burden of RR/MDR‐TB. Europe could be put into a focus of these dangerous TB strains considering that the common threshold for high TB incidence followed by most of the EU nations, which is 100 per 100,000 population, is not similar to Ukraine, which has a threshold of 73 per 100,000. This could worsen the situation as Ukrainians are not mandatory tested for TB infections because Ukraine statistically does not reach these values.^1^ Eventually, the Russia/Ukraine conflict brought about alarming public health concerns regarding TB.

## 2 TB INFECTION BEFORE AND DURING THE CONFLICT

The burden of TB in Ukraine has been chronically high even before the war; since the invasion of Russia in Ukraine, nearly 400 health facilities have been terribly destroyed, including three TB hospitals, meanwhile leaving the HCWs severely injured or killed.^4^, ^5^ The war has increased the burden of TB in Ukraine, which already had the fourth‐highest TB incidence in the European region.^6^, ^7^ In addition, following the crises in the country's health facilities upon the effects of the war, there is a great risk of an increase in the prevalence of other major health problems such as COVID‐19, TB‐HIV coinfection, and MDR TB.^7^, ^8^, ^9^ The war also has caused and contributed to spreading TB and other diseases to the countries where Ukrainian refugees flee.^8^

Before the occurrence of the conflict, cases of TB in the country spiked up to 30,000 annually while among those cases, 29% of all new diagnoses were MDR‐TB cases and only 81% of the total TB cases were diagnosed and treated. On the other hand, during early days of the conflict it was revealed that 6837 individuals were diagnosed to have TB.^10^ Following the large movement of Ukrainians migrating from their invaded country, it has been difficult for healthcare workers to diagnose and treat TB patients properly. Significantly, this has led to higher risks of transmission of the TB epidemic and other infectious diseases among the refugees and other hosting countries' citizens. Eventually, leaving no solution to the high incidences and burden of TB infection in Ukraine.^6^, ^8^

One of the catastrophic challenges caused by this conflict is the short‐time fall of the economy in Ukraine and other countries. Poverty, as one of the most potent financial determinants, may increase the risks of infection, thus causing a significant TB burden in the community affected.^11^ More than 40 million new people were reported to go below the poverty line immediately after the Russia/Ukraine conflict; as a result, health services were and are still severely affected during the war. Furthermore, the presence of other dangerous infectious diseases, such as COVID‐19, poses a great health threat aggravating health risk for the population and outweighing the capacity of health facilities to provide the services efficiently.^8^

## 3 EFFORTS

Unfortunately, the war has been a major impediment in implementing the efforts to fight TB in Ukraine. However, the WHO, EU, UK, and other global health experts are still optimistic in the fight to alleviate TB in Ukraine despite the continuing war. Refugees have been granted access to necessary healthcare, enabling access to TB and MDR TB diagnosis and continual treatment. Also, some funders have allocated emergency funds for TB services in Ukraine to help combat the TB Epidemic.^4^, ^6^

In addition, medical organizations that used to provide care for TB patients before the invasion of Ukraine continue to provide healthcare services by delivering medicines to the victims in the remote areas affected by the tragedy of the war despite the dangerous situation. However, the medical personnel find themselves partially performing their duties, fearing for their lives, so the fights continue in areas where they provide services; hence it becomes difficult to deliver healthcare services efficiently to the needy.^5^, ^6^

Testing for TB infection and screening for TB disease among refugees arriving in European countries from Ukraine during the war; There was a necessity for taking that action so as to minimize the spread of TB and this came after the European Council decided to provide temporary protection of displaced persons from Ukraine fleeing to neighboring EU Member.^1^ Moreover, the Red Cross in Kramatorsk continued to assist the victims of Ukraine−Russia conflicts in different ways to prevent the amplification of drug resistance (DR‐TB), transmission of TB infections, and abrupt death an

Big Financial organizations, such as The Global Fund, have also been contributing invaluable support to the Ukraine government by supporting the Ukrainian refugees in neighboring countries and providing funds for fostering TB and HIV services in the country.^4^ Aligning with that, Medecins Sans Frontieres, an organization that provides medical assistance to people affected by conflicts, epidemics, disasters, or exclusion from healthcare, through its team in collaboration with other hospitals across the country has pressed efforts on providing supplies needed to rescue HIV and TB programs as one of their medical activities. They offered over 170 international and more than 400 Ukrainian medical staff (doctors, nurses, surgeons) working in response to the war in Ukraine.^12^

## 4 RECOMMENDATIONS

As statistics show, Ukraine has low immunization records against MDR‐TB; there must be a close reform and third eye concern about this problem. To increase the supplies of drugs and efforts to reach the people with concerned problem WHO and other Public Health Organizations, should continue to use the main routes of transportation in Ukraine that are not highly affected by the war especially in frontline parts of war, for example the railway transportation, as Russian troops have not highly targeted the transportation route, by doing this, as many as the medical facilities will be delivered to the area especially to the highly affected parts of Ukraine including Kyiv region.

Since it's very difficult to predict the end days of the war, and for the WHO restrictions, the authorities should critically look into the screening of refugees so as to avoid stigmatization and discrimination cases against refugees.^1^ Also, regarding that, Ukraine is the second leading country in the region with the highest cases of MDR‐TB, and more than 7 million people have left the country and the threshold is 79 per 100,000 population are estimated to have the MDR‐TB, a critical concern by the authorities should be laid for the time being. WHO should call for the emergency public health concern that the refugees should be allowed to be screened to reduce the prevalence and the number of TB infections among the citizens in host countries and even healthcare workers at the refugee camps. Avoiding the initiation of treatment against TB by only considering the population at risk and vulnerable populations is vital, as the medication may cause side effects to the population given without being diagnosed for the disease.^6^

Besides that, the follow‐up for Ukrainian citizens that were continuing with the treatment has been destroyed; the communication technology techniques should also be imposed as one of the strategies to get a follow‐up with the lost Ukrainian refugees and internal scattered population under treatment, this can be done through social medias like Facebook and WhatsApp provided that the records of patients under follow‐up is there, this will help to know the location of some of patients, knowing that will help to pave down the ways that they could be reached and being assisted.^1^, ^6^

WHO, Global Health Organizations as well as NGOs that are struggling to get helping TB patients, should actively use the presence of Ukraine's national broadcasting media plus other entertainment television channels that are still broadcasting around the country to regularly announce on what health measures should the TB patients do and adhere to put themselves in health status, what people should do to protect themselves from the infection without stigmatizing the TB patients. The announcement may also show the nearest location where the concerned patient could go and get medical assistance from WHO and other healthcare experts. In addition to that, the widely used social media in the country like Instagram and Facebook, could be used to do this, by doing this the TB cases incidence in the country will be reduced.^1^, ^8^

For the sake of patients and Ukraine citizens, the Ukraine government and other countries that are helping Ukraine should foster their maximum efforts on the peace negotiations with Russia for the time being. Immediately peace will resume rapid medical reforms and programs in the country that are already destroyed, for TB and other communicable as well as non‐communicable diseases could be taken under control, for example, HIV, Covid‐19, and Diabetes.^1^, ^6^

## 5 CONCLUSION

The presence of multidrug resistant TB in Ukraine in high prevalence and the possibility of being transmitted to other people in Ukraine or neighboring countries seem to be the most feared consequence. There must be well‐prepared camps where the war refugees can be diagnosed to identify whether they have TB or not; if found positive, they must be immediately introduced to medications.^1^ For those TB patients who are still in Ukraine must be supported economically as war has left most people helpless and even those who are capable of buying drugs find no drugs available as main infrastructures for transportation of drugs and other medical equipment have been destroyed. Also, there is a shortage of electrical energy system,^9^ which is essential for running electrical medical equipment.

Therefore, it's a call toward WHO to ensure drugs are delivered and implemented for alternative sources of energy in Ukraine, and TB patients are followed‐up to see if they are adhering to prescribed medications. It is also worth appreciating the impactful developmental projects and programs that WHO initiated in Ukraine before the war started in collaboration with Ukraine and other international institutions like World Bank, which is responsible for their maintenance and monitoring. Furthermore, since most TB patients are always immunocompromised or immunodeficient, particularly due to HIV/AIDS, attacked by chronic diseases, others might be elders, women, and children, who need better nutrition assistance for better function of drugs and maintenance of their health and body immunity.

## AUTHOR CONTRIBUTIONS

**Innocent K. Paul**: Writing—review & editing. **Goodluck Nchasi**: Conceptualization; project administration; writing—review & editing. **Deusdedith B. Bulimbe**: Writing—original draft. **Meshack K. Mollel**: Writing—original draft. **George G. Msafiri**: Writing—original draft. **Alex Mbogo**: Writing—original draft. **Mayunga B. Mswanzari**: Writing—original draft. **Msengi Joseph**: Writing—original draft. **Ashraf Mahmoud**: Conceptualization; writing—review & editing. **Anastasiia Volkova**: Writing—review & editing.

## CONFLICT OF INTEREST STATEMENT

The authors declare no conflict of interest

## TRANSPARENCY STATEMENT

The lead author Innocent K. Paul affirms that this manuscript is an honest, accurate, and transparent account of the study being reported; that no important aspects of the study have been omitted; and that any discrepancies from the study as planned (and, if relevant, registered) have been explained.

## Data Availability

The authors confirm that no primary data was collected or associated with this article.
